# (4*R*,5*S*)-4-Hy­droxy­meth­yl-5-[(methyl­sulfanyl)­meth­yl]-1,3-oxazolidin-2-one

**DOI:** 10.1107/S1600536812025809

**Published:** 2012-06-13

**Authors:** Graeme J. Gainsford, Keith Clinch

**Affiliations:** a Industrial Research Limited, PO Box 31-310, Lower Hutt, New Zealand

## Abstract

The title compound, C_6_H_11_NO_3_S, crystallizes utilizing a three-dimensional set of O—H⋯O, N—H⋯O and C—H⋯O hydrogen bonds. The 1,3-oxazolidin-2-one ring adopts an envelope conformation with the C atom bearing the hy­droxy­methyl group as the flap.

## Related literature

For related structures, see Evans *et al.* (2007 ▶); Pallavicini *et al.* (2004 ▶). For the synthesis, see: Clinch *et al.* (2012 ▶). For a description of the Cambridge Structural Database, see: Allen (2002 ▶); For conformational analysis, see: Cremer & Pople (1975 ▶). For hydrogen-bond motifs, see: Bernstein *et al.* (1995 ▶).
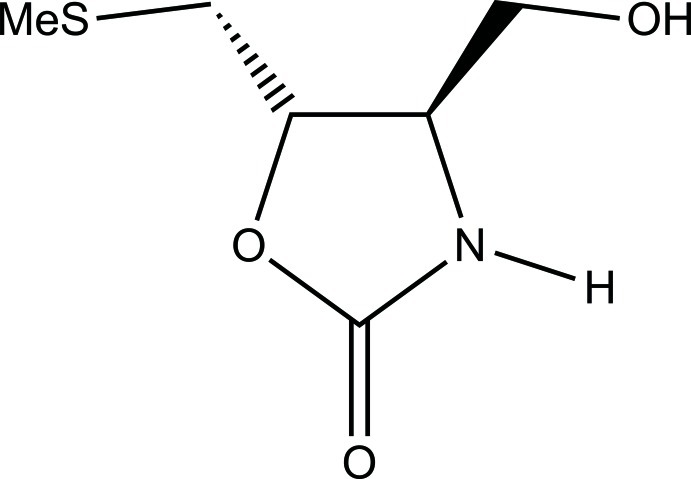


## Experimental

### 

#### Crystal data

C_6_H_11_NO_3_S
*M* *_r_* = 177.22
Monoclinic, 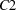

*a* = 9.7821 (4) Å
*b* = 7.9620 (3) Å
*c* = 11.5472 (4) Å
β = 109.837 (2)°
*V* = 845.99 (6) Å^3^
*Z* = 4
Mo *K*α radiation
μ = 0.34 mm^−1^
*T* = 98 K
0.45 × 0.23 × 0.07 mm

#### Data collection

Bruker–Nonius APEXII CCD area-detector diffractometer
Absorption correction: multi-scan [Blessing (1995 ▶) and *SADABS* (Sheldrick, 1996 ▶)] *T*_min_ = 0.854, *T*_max_ = 0.980
16080 measured reflections
3172 independent reflections
3091 reflections with *I* > 2σ(*I*)
*R*_int_ = 0.021

#### Refinement

*R*[*F* ^2^ > 2σ(*F* ^2^)] = 0.022
*wR*(*F* ^2^) = 0.060
*S* = 1.07
3172 reflections
142 parameters
2 restraints
All H-atom parameters refined
Δρ_max_ = 0.38 e Å^−3^
Δρ_min_ = −0.21 e Å^−3^
Absolute structure: Flack (1983 ▶), 1409 Friedel pairs
Flack parameter: 0.02 (4)

### 

Data collection: *APEX2* (Bruker, 2005 ▶); cell refinement: *SAINT* (Bruker, 2005 ▶); data reduction: *SAINT*; program(s) used to solve structure: *SHELXS97* (Sheldrick, 2008 ▶); program(s) used to refine structure: *SHELXL97* (Sheldrick, 2008 ▶); molecular graphics: *ORTEP-3* (Farrugia, 1997 ▶) and *Mercury* (Macrae *et al.*, 2008 ▶); software used to prepare material for publication: *SHELXL97* and *PLATON* (Spek, 2009 ▶).

## Supplementary Material

Crystal structure: contains datablock(s) global, I. DOI: 10.1107/S1600536812025809/im2379sup1.cif

Structure factors: contains datablock(s) I. DOI: 10.1107/S1600536812025809/im2379Isup2.hkl

Additional supplementary materials:  crystallographic information; 3D view; checkCIF report

## Figures and Tables

**Table 1:** Hydrogen-bond geometry (Å, °)

*D*—H⋯*A*	*D*—H	H⋯*A*	*D*⋯*A*	*D*—H⋯*A*
N3—H3*N*⋯O3^i^	0.829 (14)	2.029 (14)	2.8442 (9)	167.4 (14)
O3—H3*O*⋯O2^ii^	0.74 (2)	1.97 (2)	2.7018 (9)	171 (2)
C5—H5⋯O2^iii^	0.952 (13)	2.426 (14)	3.2264 (10)	141.6 (11)

Symmetry codes: (i) 
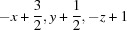
; (ii) 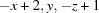; (iii) 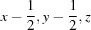.

**Fig. 1.**
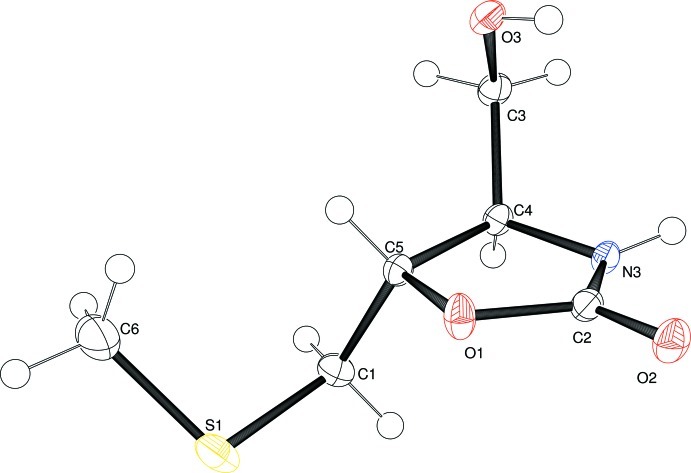
Molecular structure of the asymmetric unit of (I) (Farrugia, 1997) with 50% probabilility ellipsoids.

**Fig. 2.**
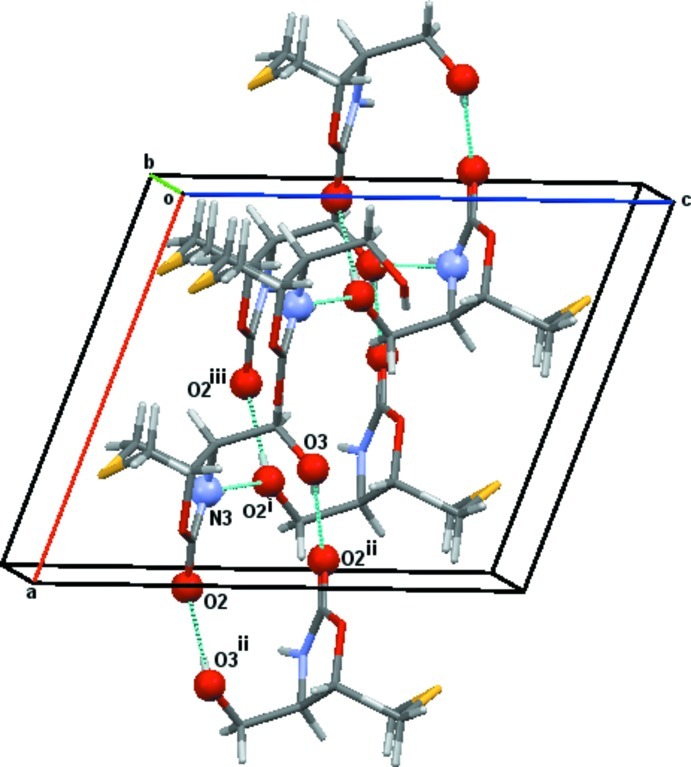
Packing diagram of the unit cell showing some key interactions (see text and Table 1) (Macrae et al., 2008). Hydrogen bond interactions are shown as blue dotted lines. Symmetry (i) 3/2 - x, 1/2 + y, 1 - z (ii) 2 - x, y, 1 - z (iii) x - 1/2, 1/2 + y, z

## supplementary crystallographic information

### Comment

This study is part of a programme aimed at preparing transition state analogue inhibitors of human methylthioadenosine phosphorylase and bacterial methylthioadenosine/S-adenosylhomocysteine nucleosidase. The title compound was produced by an unexpected rearrangement and was studied to confirm the structure and the molecular stereochemistry. The full details of the synthesis of the title compound are presented elsewhere (Clinch *et al.*, 2012).

The asymmetric unit and labelling is shown in Fig 1. The absolute stereochemistry with C4(*R*) and C5(*S*) was determined from the anomalous dispersion, with Hooft *y* value 0.028 (12). The 1,3-oxazolidin-2-one ring adopts an envelope conformation with flap atom C4 (Cremer & Pople, 1975, parameters are Q(2) 0.0980 (8) Å and φ 105.2 (5)°) with similar dimensions to the related (Allen, 2002; CSD Version 5.33, with February 2012 updates) 5-(*p*-tolylthiocarbonyl) (Evans *et al.*, 2007) and 4-hydroxymethyl-2-oxazolidine (Pallavicini *et al.*, 2004) structures.

The basic crystal packing can be described (Bernstein *et al.*, 1995) with two C(5) motifs, corresponding to entries 1 and 3 in Table 1, which provide binding parallel to the *bc* and *ab* planes, respectively. The third interaction (entry 2, Table 1) makes an *R*^2^_2_(14) motif in the *ac* plane utilizing a 2-fold axis (Figure 2). The ability of the hydroxymethyl OH group to act as both donor (through its H atom) and acceptor (to adjacent nitrogen protons) is also observed in most of the related oxazolidinone structures.

### Experimental

The preparation of the title compound is given by Clinch *et al.* (2012). Crystals were obtained by dissolving the title compound in a minimum volume of ethanol, adding hexanes until just before the turbidity point then setting aside at ambient temperature until crystallization was complete.

### Refinement

All H atoms except those on C6 were refined with isotropic thermal parameters. The O3–HO3 bond was constrained to 0.82 Å using *DFIX* and the three H atoms on the methyl atom C6 were refined with a common isotropic thermal parameter.

### Crystal data

**Table:**

C_6_H_11_NO_3_S	*F*(000) = 376
*M**_r_* = 177.22	*D*_x_ = 1.391 Mg m^−^^3^
Monoclinic, *C*2	Mo *K*α radiation, λ = 0.71073 Å
Hall symbol: C 2y	Cell parameters from 6722 reflections
*a* = 9.7821 (4) Å	θ = 3.4–34.8°
*b* = 7.9620 (3) Å	µ = 0.34 mm^−^^1^
*c* = 11.5472 (4) Å	*T* = 98 K
β = 109.837 (2)°	Plate, colourless
*V* = 845.99 (6) Å^3^	0.45 × 0.23 × 0.07 mm
*Z* = 4	

### Data collection

**Table:**

Bruker–Nonius APEXII CCD area-detector diffractometer	3172 independent reflections
Radiation source: fine-focus sealed tube	3091 reflections with *I* > 2σ(*I*)
Graphite monochromator	*R*_int_ = 0.021
Detector resolution: 8.192 pixels mm^-1^	θ_max_ = 34.8°, θ_min_ = 3.4°
phi and ω scans	*h* = −14→15
Absorption correction: multi-scan [Blessing (1995) and *SADABS* (Sheldrick, 1996)]	*k* = −11→12
*T*_min_ = 0.854, *T*_max_ = 0.980	*l* = −17→17
16080 measured reflections	

### Refinement

**Table:**

Refinement on *F*^2^	Secondary atom site location: difference Fourier map
Least-squares matrix: full	Hydrogen site location: inferred from neighbouring sites
*R*[*F*^2^ > 2σ(*F*^2^)] = 0.022	All H-atom parameters refined
*wR*(*F*^2^) = 0.060	*w* = 1/[σ^2^(*F*_o_^2^) + (0.0347*P*)^2^ + 0.1867*P*] where *P* = (*F*_o_^2^ + 2*F*_c_^2^)/3
*S* = 1.07	(Δ/σ)_max_ = 0.001
3172 reflections	Δρ_max_ = 0.38 e Å^−^^3^
142 parameters	Δρ_min_ = −0.21 e Å^−^^3^
2 restraints	Absolute structure: Flack (1983), 1409 Friedel pairs
Primary atom site location: structure-invariant direct methods	Flack parameter: 0.02 (4)

### Special details

**Table:**

Experimental. One backstop screened reflection (0,0,1) was omitted in the refinement; 1 other reflection (2,0,0) within sin(theta)/lambda of 0.5 was not collected.
Geometry. All e.s.d.'s (except the e.s.d. in the dihedral angle between two l.s. planes) are estimated using the full covariance matrix. The cell e.s.d.'s are taken into account individually in the estimation of e.s.d.'s in distances, angles and torsion angles; correlations between e.s.d.'s in cell parameters are only used when they are defined by crystal symmetry. An approximate (isotropic) treatment of cell e.s.d.'s is used for estimating e.s.d.'s involving l.s. planes.
Refinement. Refinement of *F*^2^ against ALL reflections. The weighted *R*-factor *wR* and goodness of fit *S* are based on *F*^2^, conventional *R*-factors *R* are based on *F*, with *F* set to zero for negative *F*^2^. The threshold expression of *F*^2^ > σ(*F*^2^) is used only for calculating *R*-factors(gt) *etc*. and is not relevant to the choice of reflections for refinement. *R*-factors based on *F*^2^ are statistically about twice as large as those based on *F*, and *R*- factors based on ALL data will be even larger.

### Fractional atomic coordinates and isotropic or equivalent isotropic displacement parameters (Å^2^)

**Table:**

	*x*	*y*	*z*	*U*_iso_*/*U*_eq_	
S1	0.73433 (3)	0.27672 (3)	0.07622 (2)	0.02645 (6)	
O1	0.88351 (6)	0.51738 (7)	0.30179 (6)	0.01702 (11)	
O2	1.03771 (6)	0.73322 (8)	0.37235 (6)	0.01786 (12)	
O3	0.72175 (7)	0.54658 (8)	0.52052 (6)	0.01844 (12)	
N3	0.79288 (6)	0.75917 (8)	0.33788 (6)	0.01278 (11)	
C1	0.66012 (9)	0.45196 (11)	0.13436 (8)	0.01841 (15)	
C2	0.91379 (8)	0.67743 (9)	0.34084 (7)	0.01297 (12)	
C3	0.61655 (8)	0.62734 (10)	0.41960 (8)	0.01628 (13)	
C4	0.66762 (7)	0.64864 (9)	0.30976 (7)	0.01290 (12)	
C5	0.72876 (8)	0.48658 (9)	0.27115 (7)	0.01284 (12)	
C6	0.6659 (2)	0.10352 (15)	0.14198 (13)	0.0421 (3)	
H3O	0.7827 (16)	0.606 (2)	0.5473 (14)	0.031 (4)*	
H3N	0.7992 (14)	0.8487 (18)	0.3760 (13)	0.018 (3)*	
H1A	0.5627 (16)	0.436 (2)	0.1172 (15)	0.023 (3)*	
H1B	0.6732 (17)	0.545 (2)	0.0903 (15)	0.029 (4)*	
H3A	0.5946 (14)	0.7311 (17)	0.4411 (12)	0.018 (3)*	
H3B	0.5319 (15)	0.554 (2)	0.3952 (13)	0.024 (3)*	
H4	0.5890 (16)	0.6908 (19)	0.2414 (14)	0.024 (3)*	
H5	0.7184 (13)	0.3900 (17)	0.3160 (12)	0.012 (3)*	
H6A	0.552 (2)	0.103 (3)	0.118 (2)	0.052 (3)*	
H6B	0.693 (2)	−0.003 (3)	0.1116 (19)	0.052 (3)*	
H6C	0.713 (2)	0.106 (3)	0.230 (2)	0.052 (3)*	

### Atomic displacement parameters (Å^2^)

**Table:**

	*U*^11^	*U*^22^	*U*^33^	*U*^12^	*U*^13^	*U*^23^
S1	0.03705 (12)	0.02584 (11)	0.01991 (10)	0.00124 (9)	0.01417 (8)	−0.00624 (8)
O1	0.0122 (2)	0.0129 (2)	0.0260 (3)	−0.00050 (18)	0.0066 (2)	−0.0043 (2)
O2	0.0126 (2)	0.0179 (3)	0.0234 (3)	−0.00324 (19)	0.0065 (2)	−0.0010 (2)
O3	0.0216 (3)	0.0178 (3)	0.0166 (3)	−0.0059 (2)	0.0074 (2)	0.0020 (2)
N3	0.0127 (2)	0.0100 (3)	0.0170 (3)	−0.0009 (2)	0.0068 (2)	0.0000 (2)
C1	0.0219 (3)	0.0197 (4)	0.0134 (3)	−0.0007 (3)	0.0057 (3)	−0.0012 (3)
C2	0.0136 (3)	0.0120 (3)	0.0141 (3)	−0.0008 (2)	0.0057 (2)	0.0007 (2)
C3	0.0157 (3)	0.0161 (3)	0.0205 (4)	−0.0008 (2)	0.0106 (3)	−0.0003 (3)
C4	0.0108 (2)	0.0129 (3)	0.0149 (3)	−0.0005 (2)	0.0043 (2)	0.0005 (2)
C5	0.0121 (2)	0.0127 (3)	0.0139 (3)	−0.0019 (2)	0.0047 (2)	−0.0008 (2)
C6	0.0847 (11)	0.0204 (4)	0.0279 (6)	0.0071 (5)	0.0281 (7)	0.0016 (4)

### Geometric parameters (Å, º)

**Table:**

S1—C1	1.8042 (9)	C1—H1A	0.914 (15)
S1—C6	1.8085 (14)	C1—H1B	0.930 (17)
O1—C2	1.3507 (9)	C3—C4	1.5222 (11)
O1—C5	1.4538 (9)	C3—H3A	0.909 (14)
O2—C2	1.2247 (9)	C3—H3B	0.972 (14)
O3—C3	1.4201 (11)	C4—C5	1.5499 (10)
O3—H3O	0.743 (13)	C4—H4	0.957 (15)
N3—C2	1.3402 (9)	C5—H5	0.952 (13)
N3—C4	1.4530 (9)	C6—H6A	1.06 (2)
N3—H3N	0.829 (14)	C6—H6B	0.99 (2)
C1—C5	1.5172 (11)	C6—H6C	0.97 (2)
			
C1—S1—C6	100.40 (5)	H3A—C3—H3B	111.4 (12)
C2—O1—C5	109.43 (6)	N3—C4—C3	111.81 (6)
C3—O3—H3O	107.9 (13)	N3—C4—C5	100.96 (5)
C2—N3—C4	112.43 (6)	C3—C4—C5	114.50 (6)
C2—N3—H3N	119.9 (9)	N3—C4—H4	110.7 (9)
C4—N3—H3N	123.0 (9)	C3—C4—H4	109.1 (9)
C5—C1—S1	115.86 (6)	C5—C4—H4	109.6 (9)
C5—C1—H1A	108.2 (10)	O1—C5—C1	109.90 (6)
S1—C1—H1A	109.3 (10)	O1—C5—C4	105.14 (6)
C5—C1—H1B	109.3 (10)	C1—C5—C4	111.91 (6)
S1—C1—H1B	105.2 (10)	O1—C5—H5	107.4 (7)
H1A—C1—H1B	108.7 (14)	C1—C5—H5	109.2 (8)
O2—C2—N3	127.40 (7)	C4—C5—H5	113.1 (8)
O2—C2—O1	121.61 (7)	S1—C6—H6A	113.8 (13)
N3—C2—O1	110.98 (6)	S1—C6—H6B	108.9 (12)
O3—C3—C4	112.51 (6)	H6A—C6—H6B	107.0 (17)
O3—C3—H3A	111.1 (8)	S1—C6—H6C	108.3 (13)
C4—C3—H3A	107.6 (9)	H6A—C6—H6C	111.1 (17)
O3—C3—H3B	106.0 (9)	H6B—C6—H6C	107.7 (17)
C4—C3—H3B	108.3 (8)		
			
C6—S1—C1—C5	71.48 (8)	C2—O1—C5—C1	115.03 (7)
C4—N3—C2—O2	−173.09 (8)	C2—O1—C5—C4	−5.56 (8)
C4—N3—C2—O1	7.65 (9)	S1—C1—C5—O1	58.29 (8)
C5—O1—C2—O2	179.83 (7)	S1—C1—C5—C4	174.71 (5)
C5—O1—C2—N3	−0.86 (9)	N3—C4—C5—O1	9.13 (7)
C2—N3—C4—C3	111.84 (7)	C3—C4—C5—O1	−111.14 (7)
C2—N3—C4—C5	−10.33 (8)	N3—C4—C5—C1	−110.13 (7)
O3—C3—C4—N3	−64.24 (8)	C3—C4—C5—C1	129.59 (7)
O3—C3—C4—C5	49.80 (9)		

### Hydrogen-bond geometry (Å, º)

**Table:**

*D*—H···*A*	*D*—H	H···*A*	*D*···*A*	*D*—H···*A*
N3—H3*N*···O3^i^	0.829 (14)	2.029 (14)	2.8442 (9)	167.4 (14)
O3—H3*O*···O2^ii^	0.74 (2)	1.97 (2)	2.7018 (9)	171 (2)
C5—H5···O2^iii^	0.952 (13)	2.426 (14)	3.2264 (10)	141.6 (11)

Symmetry codes: (i) −*x*+3/2, *y*+1/2, −*z*+1; (ii) −*x*+2, *y*, −*z*+1; (iii) *x*−1/2, *y*−1/2, *z*.
